# Free radical scavenging and formation by multi-walled carbon nanotubes in cell free conditions and in human bronchial epithelial cells

**DOI:** 10.1186/1743-8977-11-4

**Published:** 2014-01-18

**Authors:** Penny Nymark, Jensen Keld Alstrup, Satu Suhonen, Yahia Kembouche, Minnamari Vippola, Jos Kleinjans, Julia Catalán, Hannu Norppa, Joost van Delft, Jacob Jan Briedé

**Affiliations:** 1 Department of Toxicogenomics, Maastricht University, Maastricht, The Netherlands; 2 Danish Centre for Nanosafety, National Research Centre for the Working Environment, Copenhagen, Denmark; 3 Nanosafety Research Centre and Systems Toxicology, Finnish Institute of Occupational Health, Helsinki, Finland; 4 Department of Materials Science, Tampere University of Technology, Tampere, Finland; 5 Department of Anatomy, Embriology and Genetics, University of Zaragoza, Zaragoza, Spain

**Keywords:** Asbestos, Electron Spin Resonance, Free radicals, Glass wool, Human bronchial epithelial cells, Multi-walled carbon nanotubes

## Abstract

**Background:**

Certain multi-walled carbon nanotubes (MWCNTs) have been shown to elicit asbestos-like toxicological effects. To reduce needs for risk assessment it has been suggested that the physicochemical characteristics or reactivity of nanomaterials could be used to predict their hazard. Fibre-shape and ability to generate reactive oxygen species (ROS) are important indicators of high hazard materials. Asbestos is a known ROS generator, while MWCNTs may either produce or scavenge ROS. However, certain biomolecules, such as albumin – used as dispersants in nanomaterial preparation for toxicological testing *in vivo* and *in vitro* - may reduce the surface reactivity of nanomaterials.

**Methods:**

Here, we investigated the effect of bovine serum albumin (BSA) and cell culture medium with and without BEAS 2B cells on radical formation/scavenging by five MWCNTs, Printex 90 carbon black, crocidolite asbestos, and glass wool, using electron spin resonance (ESR) spectroscopy and linked this to cytotoxic effects measured by trypan blue exclusion assay. In addition, the materials were characterized in the exposure medium (e.g. for hydrodynamic size-distribution and sedimentation rate).

**Results:**

The test materials induced highly variable cytotoxic effects which could generally be related to the abundance and characteristics of agglomerates/aggregates and to the rate of sedimentation. All carbon nanomaterials were found to scavenge hydroxyl radicals (^•^OH) in at least one of the solutions tested. The effect of BSA was different among the materials. Two types of long, needle-like MWCNTs (average diameter >74 and 64.2 nm, average length 5.7 and 4.0 μm, respectively) induced, in addition to a scavenging effect, a dose-dependent formation of a unique, yet unidentified radical in both absence and presence of cells, which also coincided with cytotoxicity.

**Conclusions:**

Culture medium and BSA affects scavenging/production of ^•^OH by MWCNTs, Printex 90 carbon black, asbestos and glass-wool. An unidentified radical is generated by two long, needle-like MWCNTs and these two CNTs were more cytotoxic than the other CNTs tested, suggesting that this radical could be related to the adverse effects of MWCNTs.

## Background

Carbon nanotubes (CNTs) are among the most important materials in nanotechnology. Recently, the global production capacity of CNTs was estimated to exceed several thousand tons/year [1]. CNTs are added to composites of plastic and rubber to make them lighter and stronger for use in various products such as vehicles, wind turbines and sports equipment, but they can also be found in lithium-ion batteries of mobile phones and laptops, as well as in paints. Future CNT-based technology is expected to have a tremendous impact on the development of new therapeutics, building materials, electronics, energy systems, and textiles [1].

The increased use of CNTs and strong indications of high hazard of some CNTs calls for improvement in the understanding of the physicochemical differences between the test materials and hypothesis-driven toxicity testing. Some of the concerns about the hazards of CNTs are related to their high persistence and fibrous-like morphology, which is comparable to that of asbestos. However, existing toxicity data are scanty and inconsistent. Currently, most CNTs are classified as single-walled, double-walled and multi-walled CNTs (SWCNTs, DWCNTs and MWCNTs), but all of these groups include materials with great variation in size, chemical modification or functionalization, and it would be almost impossible to thoroughly test them all for toxicity. Subtle differences in physical properties and surface chemistry of the CNTs may have a large impact on their toxicity. Therefore, physicochemical characterization of CNTs tested for toxicity, has become important as reviewed by Liu et al. [2]. As stated by Fenoglio et al. [3], knowledge about physicochemical characteristics associated with adverse cellular responses is a key step in the prediction of hazard by new nanomaterials and also for the development of biocompatible ones. Such studies may reveal health effect-associated characteristics that can be used as indicators of toxicity when assessing other nanomaterials.

The potential similarities between some CNTs and asbestos were pointed out already in 1998 [4], and the first reports on the harmful effects of CNTs to animals and cells appeared almost a decade ago [5,6]. More recent data have suggested that especially long, needle-like MWCNT (MWCNT_LNL_) are able to induce asbestos-like effects both *in vivo* and *in vitro*[7-13]. Studies administering MWCNT_LNL_ (specifically Mitsui MWCNT-7) intrapleurally, intraperitoneally, intrascrotally or by inhalation to rodents have described pathological responses similar to those observed following asbestos exposure, i.e. inflammation, fibrosis and mesothelioma induction [9,11,12,14]. On the other hand, short MWCNTs did not induce inflammation or fibrosis in mice after intrapleural injection or aspiration into the lungs [14,15]. Nagai et al. (2011) observed that MWCNT_LNL_ with a diameter of about 50 nm entered mesothelial cells *in vitro* by piercing their membrane, were more toxic to cultured human mesothelial cells and induced - after intraperitoneal injection - more inflammation, fibrosis, and mesothelioma in rats, than thicker (diameter ~145 nm) and thinner tangled (diameter ~15 nm) MWCNTs; the latter material was not taken up by mesothelial cells, showed very low toxicity *in vitro*, and did not induce mesothelioma. The 50-nm MWCNT_LNL_ were suggested to induce inflammation and tumours through direct mesothelial cell injury [16,17].

Several mechanisms of toxicity, similar to the ones linked to asbestos-exposure, have been proposed for CNTs, such as (i) association of fibres with the cell membrane causing physical damage and cell membrane malfunction, (ii) protein-fibre interaction inhibiting protein function, and (iii) induction of reactive oxygen species (ROS) either directly by the CNTs themselves or indirectly through mitochondrial dysfunctions or NADPH oxidase activation induced by so-called frustrated phagocytosis in e.g. macrophages [2,18-20]. It seems probable that a combination of different mechanisms could contribute to the toxicity of CNTs, as has been considered to be the case with asbestos [21].

Asbestos is well known to be an efficient catalyst of free radicals, especially hydroxyl radicals (^•^OH), both in cell-free and cellular systems, possibly due to its high content of iron [22]. In contrast to the effects of asbestos, a few studies have indicated that some MWCNTs are efficient scavengers of ^•^OH and superoxide (^•^O_2_^-^) radicals in cell-free conditions [23]. The scavenging of free radicals by CNTs was suggested to be related to the amount and nature of defects in the CNTs, i.e. ruptures of the graphene framework [23]. In contrast, ROS formation by SWCNTs was observed in cell media with and without FE1-Muta™ Mouse lung epithelial cells, at intermediate levels between that of Printex 90 and C60 fullerene and correlated with the order of genotoxicity [24]. This type of research is still at an early stage and more thorough studies are needed as reviewed by Liu et al. [2].

CNT in powder state tend to exist as aggregates and agglomerates. The aggregation is mainly a characteristic related to the manufacturing process, where CNTs may grow parallel from a catalyst support or entangle during gas-suspended growth assisted by a floating catalyst. These characteristics, in addition to the hydrophobic nature of at least pristine CNTs make them poorly dispersible in e.g. water and simple saline solutions. Therefore, different surfactant additives have been employed to increase the dispersibility of nanomaterials in various toxicological studies. One of the most frequently used surfactant biomolecules is albumin. Bovine serum albumin (BSA) has been shown to improve dispersion of nanomaterials in several studies [25-27] and has been applied in larger harmonized studies on nanomaterial genotoxicity [28]. Thus, the influence of BSA on MWCNT-induced radical formation/scavenging needs to be studied more thoroughly. Increased ROS formation has for example been reported with and without human monocytic cells in the presence of BSA by carbon black [29].

Here, we investigated free radical formation by BSA- and non-BSA-dispersed long needle-like (two different types MWCNT_LNL1_ and MWCNT_LNL2_), long, tangled (MWCNT_LT_), short, purified (MWCNT_SP_), and short, non-purified (MWCNT_SNP_) MWCNTs as well as Printex 90 carbon black, crocidolite asbestos and glass wool (MMVF-10; see Table 1 for material characteristics) in cell-free and cellular settings using human bronchial epithelial BEAS 2B cells and electron spin resonance (ESR) spectroscopy in combination with a spin trapping technique for radical detection and identification. The results were correlated with cytotoxicity and physicochemical characterization of the materials in exposure medium. Being a carcinogenic and ROS-inducing fibre, asbestos was used as a positive control, while glass wool, which has been classified as a non-carcinogenic fibre by IARC [30], was used as negative fibre control. Carbon black was included as a non-fibrous (spherical) carbon nanomaterial control [24,29,31]. MWCNT_LNL1_ (i.e. Mitsui MWCNT-7, Table 1) was chosen because it has previously been shown to induce asbestos-like pathogenic effects as mentioned above [8-12], while MWCNT_LT_ and MWCNT_SP_ seem to be less potent as concerns asbestos-like pathogenicity and immunotoxicity *in vitro*[8,9,14]. Also, pulmonary inflammation induced by MWCNT_SP_ has been shown to regress to the level of the control over time following inhalation in mice [32]. MWCNT_LNL2_ and MWCNT_SNP_ were included due to morphological similarities to the materials described above.

**Table 1:** Test material information

**Material**	**Type**	**Name**	**Producer/distributor**	**Average diameter (nm)**^**a**^	**Average length (μm)**^**a**^	**Major elements**^**b**^	**Minor elements**^**c**^	**Specific surface area (m**^**2**^**/g)**^**d**^
Asbestos	Crocidolite	-	UICC	180	4.6	Fe, Si, Na, Mg, Ca, O		8.3^e^
Glass wool	Insulation fibre	MMVF10/Manville 901	Johns Manville, Denver, CO, USA	1100 ± 500	21 ±18	Si, Al, O, Na, Mg, K, Ca		1.07^f^
MWCNT_LNL1_	Long, needle-like	MWCNT-7	Mitsui & co, Ltd, Tokyo, Japan	74 ± 28	5.7 ± 3.7	C	< 0.5 wt% Na, Fe, Al, Mg, Ni	22 (29)
MWCNT_LNL2_	Long, needle-like	NM-401	JRC, European Commission	64.2 ± 34.5	4.0 ± 2.4	C, residues of Si	< 0.6 wt% Na, Fe, Al, Ni, Mg	18 (31)
MWCNT_LT_	Long, tangled	MWCNT 8-15 nm OD	Cheap Tubes Inc, Brattleboro, VT, USA	17 ± 7	0.5 ± 0.3	C residues of Ni, Fe	< 5 wt% Ni, Na, Fe, Al, Mg, Mn	75 (117)
MWCNT_SP_	Short, purified	Baytubes C 150 HP	Bayer Material Science, Leverkusen, Germany	12.0 ± 7.0	0.4 ± 0.2	C, residues of Si, Co	< 3 wt% Mn, Mg, Al, Na, Ni, Fe	*ND* (189)
MWCNT_SNP_	Short, non-purified	NM-400	JRC, European Commission	13.6 ± 3.7	0.8 ± 0.4	C, O, Si, Fe, Mg, Na	< 10 wt% Al, Fe, Na, Ni	254 (189)
Carbon black	Nano-sized	Printex 90	Evonik Industries AG, Essen, Germany	14	-	C, residues of Si	< 1 wt N, H^e^	295-338^g^

*ND* – not determined.

^a^For MWCNTs as detected in [33]; for other materials according to producer/distributer.

^b^Elemental analysis by TEM/EDS.

^c^From [33] - ICP-MS analysis; in order of abundance from highest concentration to lowest.

^d^From [33] – BET analysis (SAXS analysis), except for asbestos, glass wool and carbon black.

^e^From [34].

^f^From [35].

^g^From [36].

## Results and discussion

### Cytotoxicity

**Figure 1:**
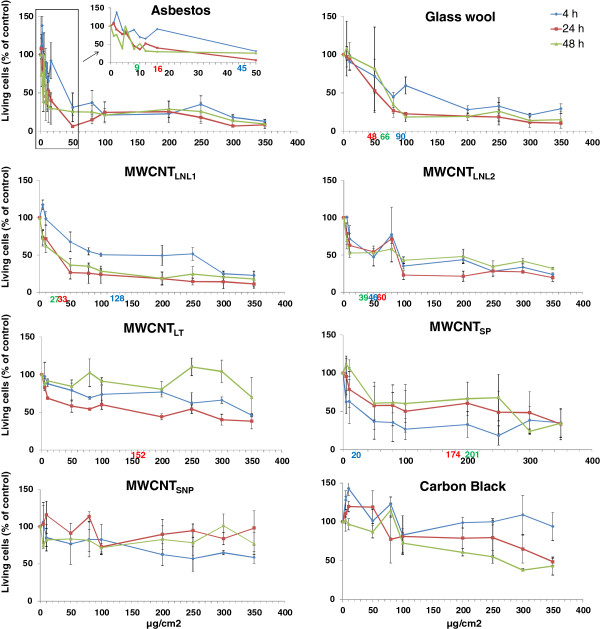
**Cytotoxicity of asbestos, glass wool, MWCNT**_**LNL1**_**, MWCNT**_**LNL2, **_**MWCNT**_**LT**_**, MWCNT**_**SP **_**and MWCNT**_**SNP **_**and carbon black in BEAS 2B cells (trypan blue exclusion assay).** The number of living cells is expressed as percentage of the number of living cells in control cultures. Symbols represent means (4 replicates ± SE). IC_50_ (as defined by fitting the data to a logarithmic trend line) is indicated by numbers in respective colour on the x- axis.

The trypan blue exclusion assay showed cytotoxicity (IC_50_) after the 4-h exposure in the following order: MWCNT_SP_ > asbestos > MWCNT_LNL2_ > glass wool > MWCNT_LNL1_ (Figure 1; Table 2). Treatment with MWCNT_LT_, MWCNT_SNP_ and carbon black did not result in 50% cytotoxicity at any dose tested. The 24-h exposure showed the following order of cytotoxicity: asbestos > MWCNT_LNL1_ > glass wool > MWCNT_LNL2_ > MWCNT_LT_ > MWCNT_SP_, while IC_50_ was not reached at any dose tested of MWCNT_SNP_ or carbon black. Finally, the 48-h exposure yielded IC_50_ values in the following order asbestos > MWCNT_LNL1_ > MWCNT_LNL2_ > glass wool > MWCNT_SP_. The other three materials (MWCNT_LT_, MWCNT_SNP_ and carbon black) did not reach 50% cytotoxicity at 48 h. Thus, considering all treatment times, asbestos was the most cytotoxic followed by MWCNT_LNL1_ and MWCNT_LNL2_. Asbestos and MWCNT_LNL1_ both showed treatment-time dependent increase in toxicity, while MWCNT_LNL2_ did not show a clear time-dependent trend, although the 48-h treatment was the most cytotoxic (Table 2). Glass wool was also clearly cytotoxic, but there was no clear dependence on treatment time. MWCNT_SP_, had the highest acute cytotoxicity (at 4 h) which, however, decreased with increasing treatment-time (Table 2). Cytotoxicity was low for MWCNT_LT_ and MWCNT_SNP_, and carbon black did not reach IC_50_ at any treatment time.

**Table 2:** **Cytotoxicity (IC**_**50**_**) of the materials**

**Material**	**IC**_**50 **_**concentration**^**a **^**(μg/cm**^**2**^**)**		
	**4 h**	**24 h**	**48 h**
Asbestos	45	16	9
Glass wool	90	48	66
MWCNT_LNL1_	128	33	27
MWCNT_LNL2_	46	60	39
MWCNT_LT_	-^b^	152	-^b^
MWCNT_SP_	20	174	201
MWCNT_SNP_	-^b^	-^b^	-^b^
Carbon black	-^b^	-^b^	-^b^

^a^Defined by fitting the data to a logarithmic trend line.

^b^IC_50_ not reached within the tested dose range.

### Physicochemical characterization of the materials

**Figure 2:**
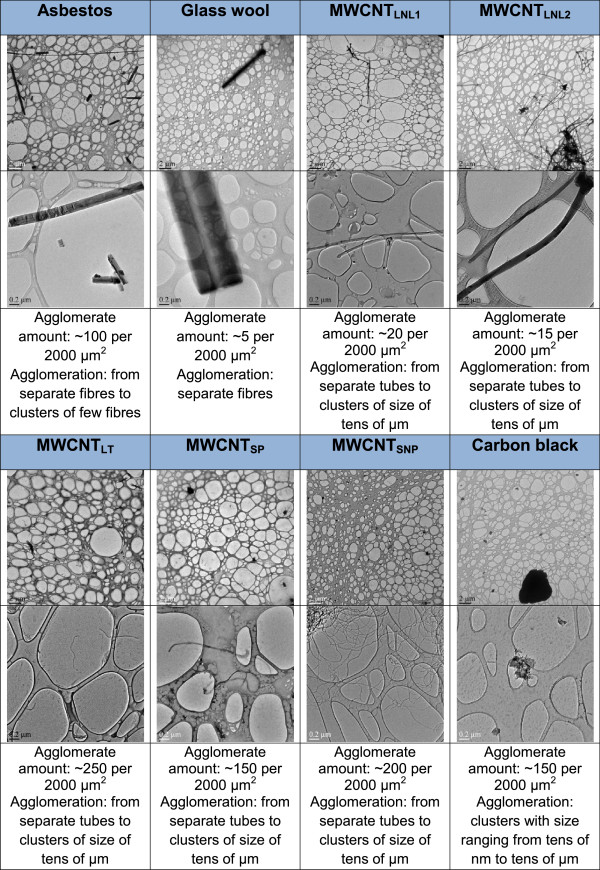
**Transmission electron microscopy (TEM) analysis on nanomaterials (1330 μg/ml, corresponding to 350 μg/cm**^**2**^**) in exposure medium (BEGM + 0.6 mg/ml BSA).** Dispersions are shown in two magnifications (the measure bar is 2 μm in the upper images and 0.2 μm in the lower images).

Material characterization by optical and transmission electron microscopy (TEM), and dynamic light scattering (DLS) showed that both single fibres/nanotubes and larger agglomerates (>10 μm) were present in all the exposure dispersions (Figure 2, and Additional file 1: Figure S1-S8). Highly separated fibres with very wide length spans were present in experiments with asbestos and glass-wool (Additional file 1: Figures S1 and S2). The longest fibres exceeded 100 μm in both asbestos (maximum observed length: ca. 800 μm) and glass-wool (maximum observed length: ca. 200 μm). A high fraction of free fibre-like CNTs was also present in the dispersions of MWCNT_LNL1_ and MWCNT_LNL2_ which, however, also contained large (up to about 100 μm-size), open-structured (optically partially transparent) agglomerates/aggregates (Additional file 1: Figures S3 and S4). Large (50 – 100 μm size) and dense (optically opaque) aggregates/agglomerates were dominant in dispersions with MWCNT_LT_, MWCNT_SNP_, and carbon black (Additional file 1: Figures S5, S7, and S8), whereas smaller dense aggregates (< 20 μm-size) were characteristic of MWCNT_SP_, (Additional file 1: Figure S6). Smaller aggregates may be internalized by the cells more efficiently, possibly explaining the acute cytotoxicity observed at 4 h. Over time the aggregates may grow in size and the cells may not be able to internalize them, enabling them - the cells - to recover and continue to grow, explaining the lower cytotoxicity at 24 and 48 h. Quantitative assessment of the small aggregate/agglomerate frequency determined by TEM showed 100-250 agglomerates/aggregates per 2000 μm^2^ in MWCNT_LT_ > MWCNT_SNP_ > MWCNT_SP_ ≈ Carbon black > Asbestos and 5-20 agglomerates/aggregates per 2000 μm^2^ in MWCNT_LNL1_ > MWCNT_LNL2_ > Glass-wool (Figure 2). However, despite the presence of these aggregates/agglomerates, a significant number of free fibres and CNTs were observed in all fibre and CNT dispersions (Figure 2).

The large primary particles and aggregates/agglomerates contributed significantly to the initial sedimentation in the *in vitro* tests. Stationary sedimentation analysis for up to 48 h using the variation in relative scattered light intensity in dynamic light scattering (DLS), suggested rapid sedimentation in dispersions with asbestos, glass-wool, and all MWCNTs, except MWCNT_SP_ (in the apparent general order MWCNT_LNL2_ > MWCNT_LNL1_ > MWCNT_SNP_ ≈ MWCNT_LT_ > > MWCNT_SP_). Particularly rapid sedimentation was seen with asbestos, MWCNT_LNL1_ and MWCNT_LNL2_ (Additional file 1: Figures S1, S3 and S4). However, in all cases the sedimentation left smaller fibres/CNT/agglomerates in the suspensions; these fibres may gradually settle at a later stage during the experiment. Thus, based on the physicochemical characteristics of the exposure suspensions, it appears that materials with a high abundance of large (up to 100 μm-size), open-structured aggregates/agglomerates and very high sedimentation rates also have strong cytotoxic effects. This suggests that a physical contact between the test material and the cells is important for the cytotoxic effects to manifest in vitro. Alternatively, the greater abundance of the singlet tubes remaining in suspension may be the source of toxicity for MWCNT_LNL1_ and MWCNT_LNL2_. Further experiments using systems, where physical contact between large agglomerates in the exposure material and the cells is prevented could provide additional answers.

### Cell-free radical formation

**Figure 3:**
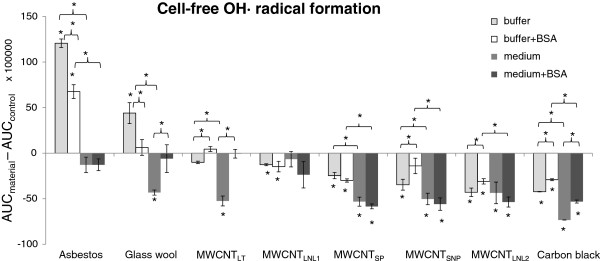
**Intensity of cell-free **^**•**^**OH formation/scavenging by asbestos, glass wool, MWCNT**_**LNL1**_**, MWCNT**_**LT**_**, MWCNT**_**SP**_**, MWCNT**_**LNL2**_**, MWCNT**_**SNP**_**, and carbon black in buffer, buffer with BSA, medium and medium with BSA as compared with respective controls.** The order of the materials is according to level of ^•^OH formation/scavenging in buffer. Columns represent the peak surface, i.e. area under curve (AUC), obtained by double integration of the DMPO peaks induced by the materials (AUC_material_) after subtracting that of the control (AUC_control_; ±SD). Statistically significant changes and differences are indicated by an asterisk (*P* < 0.05).

In order to assess hydrogen peroxide (H_2_O_2_)-induced free radical formation, cell-free ESR spectroscopy was performed on all eight materials at 1 mg/ml in buffer and bronchial epithelial growth medium (BEGM) and in buffer and BEGM supplemented with 0.6 mg/ml BSA. The samples were all compared with their respective controls, i.e. buffer or BEGM with or without BSA. Complete sample traces of each material in the four different dispersions can be seen in the supplemental material (Additional file 1: Figure S9). ^•^OH formation was the highest with asbestos in buffer with and without BSA, while glass wool induced much less ^•^OH in buffer and no ^•^OH production in buffer with BSA (Figure 3). This is consistent with the hypothesis that the carcinogenic properties of asbestos are in part associated with its ability to produce ROS [22]. It is particularly interesting to note that there was no production of radicals in combination with BSA by glass wool, a reportedly non-carcinogenic fibre [30]. In contrast, asbestos is still able to produce ROS in the presence of BSA, indicating that it could also be reactive in the human body, despite contact with proteins. Hence, together with the biodurability of crocidolite asbestos, this reactivity may add to the long-term toxic potential of the material. Remarkably, however, there was no increase of H_2_O_2_-induced radicals with asbestos in pure BEGM or with asbestos or glass wool in BEGM with BSA, indicating that both the culture medium and BSA may exhibit antioxidant properties. Indeed, albumin is known to have free-radical trapping properties [37]. In medium without BSA, glass wool had a significant scavenging effect as compared with the control level (Figure 3).

All carbon nanomaterials were found to scavenge the induction of ^•^OH in at least one of the tested solutions, which is in agreement with previous studies [3]. MWCNT_LT_ induced a significant scavenging effect in medium without BSA, but not in the other solutions, while MWCNT_LNL1_ scavenged only in buffer, both with and without BSA (Figure 3). MWCNT_SNP_ scavenged radical formation in all solutions, except in buffer with BSA. Carbon black, MWCNT_SP_ and MWCNT_LNL2_ showed the strongest scavenging ability, which could be seen in all four solutions. BSA reduced the scavenging effects of MWCNT_LT_ and carbon black in both medium and buffer and of MWCNT_LNL2_ and MWCNT_SNP_ in buffer. In general, scavenging by all nanomaterials was stronger in medium than in buffer, regardless of BSA, except for MWCNT_LNL1_ which showed a low but significant scavenging effect only in buffer (Figure 3).

**Figure 4:**
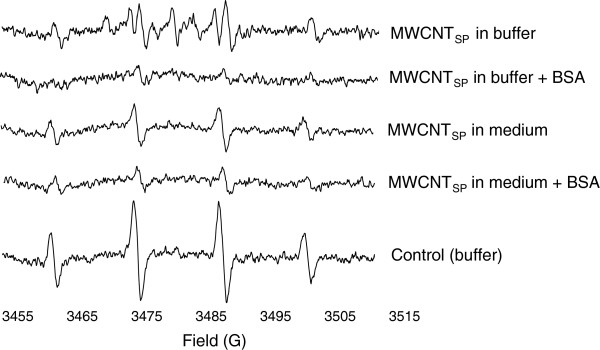
**Electron spin resonance (ESR) spectra of 1 mg/mL MWCNT**_**SP **_**in buffer (showing the induction of O**_**2**_ **·** ^**-**^**), buffer with BSA, medium and medium with BSA, and the control (buffer) in cell free settings.** Spectra are shown at identical intensity scale.

In addition to ^•^OH, some of the tested MWCNTs showed the induction of other types of radicals. For example, MWCNT_SP_ in buffer showed, based on the peak pattern and splitting constants, the formation of ^•^O_2_^-^, which could, however, not be seen with the addition of BSA or in culture medium (Figure 4). Direct ^•^O_2_^-^ production by SWCNTs has previously been detected by ESR spectrometry, and ^•^O_2_^-^ has been shown to be formed in normal and malignant human mesothelial cells following exposure to SWCNTs and crocidolite asbestos [38,39].

**Figure 5:**
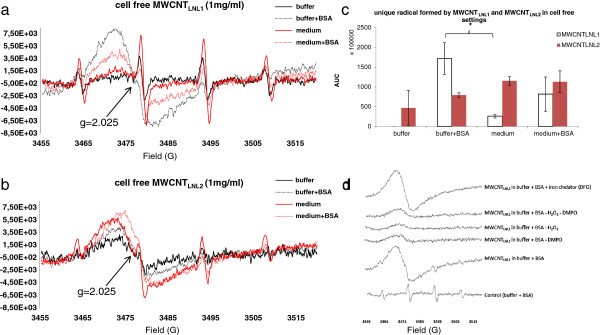
**ESR spectra in cell-free settings by 1 mg/ml a) MWCNT**_**LNL1 **_**and b) MWCNT**_**LNL2 **_**in buffer, buffer with BSA, medium and medium with BSA.** The unique peak has a g-value of 2.025 and half-width of 1.7 mT. **c)** Intensity of cell-free unique radical formation by MWCNT_LNL1_ and MWCNT_LNL2_ in buffer, buffer with BSA, medium, and medium with BSA. Columns represent the peak surface, i.e. area under curve (AUC), obtained by double integration of the unique peak (±SD). Statistically significant differences are indicated by an asterisk (*P* < 0.05). **d)** ESR spectra by MWCNT_LNL1_ in cell-free settings (in buffer with BSA) with and without DMPO and/or H_2_O_2_, and with the iron chelator deferoxamine (DFO). Spectra are shown at identical intensity scale.

The presence of BSA or culture medium also led to the formation of a yet unidentified unique radical by MWCNT_LNL1_ and the same radical could be seen by MWCNT_LNL2_ in all solutions, including buffer without BSA (Figure 5a, b and c). For MWCNT_LNL1_, the intensity of the radical was the strongest in buffer with BSA and in the presence of hydrogen peroxide (H_2_O_2_) and the spin trapping agent DMPO (Figure 5a and d). However, it could also be seen, at a lower intensity, without the addition of H_2_O_2_ and DMPO, indicating that it was formed by the nanotubes themselves and that it was a fairly stable radical (Figure 5d). The radical was not scavenged by the addition of an iron chelator, deferoxamine (DFO), indicating that it was not attributable to the iron impurity content in the samples (Table 1). In a recent review by Shvedova et al (2012) such relatively stable free radicals or radical intermediates present on the reactive surfaces of nanomaterials, including CNTs, were suggested to be involved in oxidative stress mechanisms in exposed cells [19]. Two previous studies have shown similar ESR spectra by non-purified iron-rich (26 wt%) SWCNTs, and by graphenic nanoparticles from combustion [40,41]. However, the iron-rich SWCNTs showed a g-value of 2.0 and a half-width of 64 mT, which was attributed to high-spin Fe^3+^ in a distorted tetrahedral environment [41]. The unique radical observed in our study showed a g-value of 2.025 and a half-width of 1.7 mT and the use of the iron chelator DFO showed that it was not caused by Fe^3+^ (Figure 5d). Furthermore, the iron content of both MWCNT_LNL1_ and MWCNT_LNL2_ is known to be in the order of 0.3-0.4 wt%, suggesting a minor effect due to catalyst impurities in these samples (Table 1) [33,42]. Also, other transition metal impurities were low in both these MWCNTs (< 0.6 wt%; Table 1). In the study on the graphenic nanoparticles, the authors identified an ESR signal with a much lower g-value than here, g = 2.003. This signal was attributed to a carbon-centred radical, assumed to result from the highly defective structure of the nanoparticles [40].

**Figure 6:**
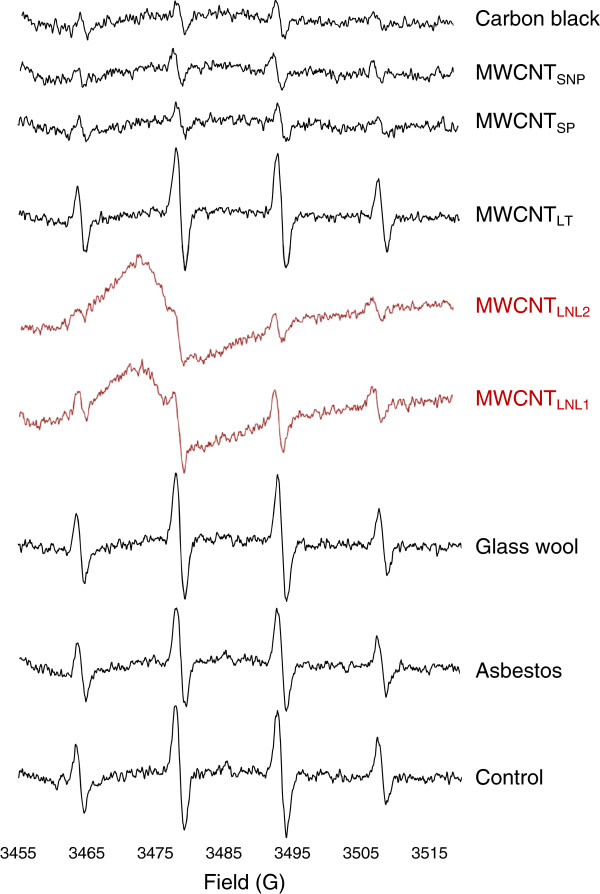
**ESR spectra in cell-free settings of control (medium with BSA), 1 mg/mL asbestos, glass wool, MWCNT**_**LNL1**_**, MWCNT**_**LNL2**_**, MWCNT**_**LT**_**, MWCNT**_**SNP**_**, MWCNT**_**SP **_**and carbon black in medium with BSA.** Spectra are shown at identical intensity scale.

None of the other materials, of which some had relatively high amounts of transition metal impurities (up to 10 wt%; Table 1.), induced a similar type of spectrum in any of the solutions as represented by dispersions in culture medium with BSA in Figure 6 (complete sample traces of all materials in each test solution can be seen in Additional file 1: Figure S9). Thus, taken the strong cytotoxicity of the two long, needle-like MWCNTs compared with the other three short or tangled MWCNTs, we speculated whether the unique radical was related to the cytotoxicity of these two MWCNT materials. Possible future studies using e.g. membrane systems to prevent physical contact between the cells and the exposure material, may provide further information as to whether the radical formation alone is indeed related to the cytotoxicity. However, the major challenge with such studies is to create a system where the radicals produced do not react before entering the test vial.

### Cellular radical formation

**Figure 7:**
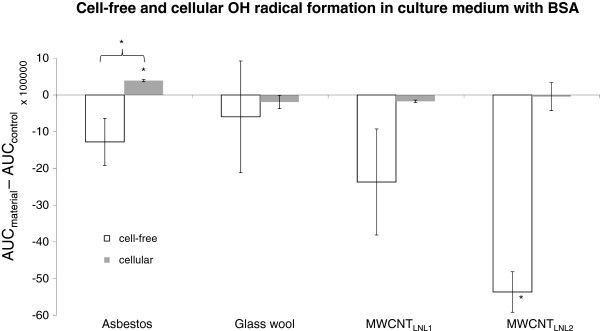
**Intensity of cell-free (1 mg/mL) and cellular (10 μg/cm**^**2**^**) **^**•**^**OH radical formation/scavenging in medium + 0.6 mg/ml BSA by asbestos, glass wool, MWCNT**_**LNL1 **_**and MWCNT**_**LNL2**_**.** Columns represent the peak surface, i.e. area under curve (AUC), obtained by double integration of the DMPO peaks induced by the materials (AUC_material_) after subtracting that of the control (AUC_control_; ±SD). Statistically significant changes are indicated by an asterisk (*P* < 0.05).

To investigate free radical formation by asbestos, glass wool, MWCNT_LNL1_ and MWCNT_LNL2_ in the presence of cells, BEAS-2B cells were first exposed to 2 and 10 μg/cm^2^ asbestos (IC_100_ and, at 4 h, IC_80_) for 30 min, 1 h, 2 h and 4 h, to identify the exposure time inducing the highest level of radicals. Thirty minutes of exposure to 10 μg/cm^2^ of asbestos induced the highest formation of ^•^OH (data not shown). Thus, cells were exposed to asbestos and glass wool (10 μg/cm^2^), as well as MWCNT_LNL1_ and MWCNT_LNL2_ (10, 20, 40 and 80 μg/cm^2^) in medium with BSA for 30 min. A slight but significant level of ^•^OH was produced by asbestos in the presence of cells and BSA, while glass wool did not cause an induction of ^•^OH at 10 μg/cm^2^. This is interesting, considering that asbestos did not show any formation of ^•^OH in medium with BSA in the cell-free settings, indicating that the radicals in this case were produced by the cells due to the exposure (Figure 7).

**Figure 8:**
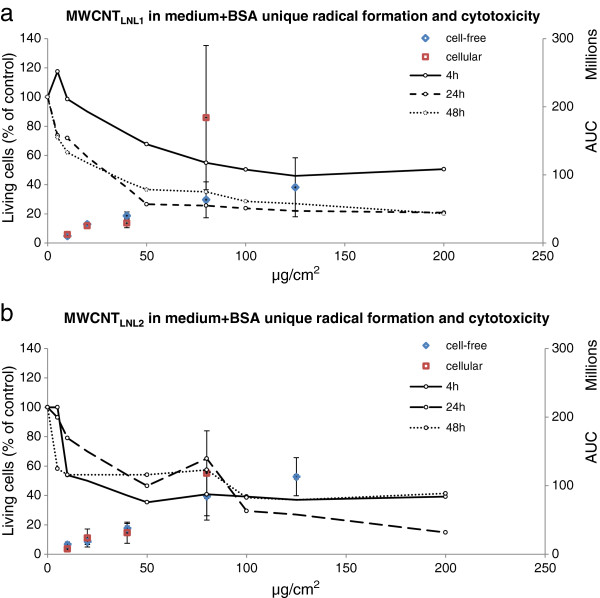
**Cytotoxicity of a) MWCNT**_**LNL1 **_**and b) MWCNT**_**LNL2 **_**plotted against the intensity of the unique radical formed by MWCNT**_**LNL1 **_**and MWCNT**_**LNL2 **_**in medium + 0.6 mg/ml BSA in cell-free (80, 160, 320, 640 and 1000 μg/ml, representing 10, 20, 40, 80 and 125 μg/cm**^**2**^**) and cellular settings (80, 160, 320 and 640 μg/ml, representing 10, 20, 40 and 80 μg/cm**^**2**^**).** The dose 10 μg/cm^2^ (80 μg/ml) was not tested in cell-free settings, and 1000 μg/ml (125 μg/cm^2^) was not tested in the cells. Columns represent the peak surface, i.e. area under curve (AUC), obtained by double integration of the unique peak (±SD). Lines represent cytotoxicity results (0-200 μg/cm^2^, representing 0-760 μg/ml). The average number of living cells is expressed as percentage of the number of living cells in control cultures. Symbols represent means. *P* < 0.001 (cell-free) and *P* < 0.0001 (cellular) for dose-dependent formation of the unique radical by both MWCNT_LNL1_ and MWCNT_LNL2_.

The formation of the unique radical induced by both MWCNT_LNL1_ and MWCNT_LNL2_ in cell free conditions, could also be seen in the presence of cells and the intensity of the radical increased dose-dependently at 10-80 μg/cm^2^ (*P* < 0.001). Furthermore, the dose-dependent formation of the radical coincided with the cytotoxicity of both materials (Figure 8). Doses below 10 μg/cm^2^ did not show the induction of the unique radical (data not shown), while doses above 80 μg/cm^2^ were not tested, due to high cytotoxicity (40%) after 4 h of exposure in the trypan blue exclusion assay (Figure 1). Consequently, the respective doses, 10, 20, 40 and 80 μg/cm^2^ (i.e. 80, 160, 320 and 640 μg/ml) were also tested in cell-free conditions, and similar results were obtained (*P* < 0.0001 for dose-dependent increase; Figure 8). At the highest dose, a higher intensity of the unique radical could be seen in the cellular settings, but the variance was very high between the replicates and the difference was not statistically significant. MWCNT_LNL1_ was also studied in the cells at 40 μg/cm^2^, in only medium and without the addition of the spin trap DMPO. The radical could be seen in all settings, consistent with the findings in the cell-free settings (data not shown).

**Figure 9:**
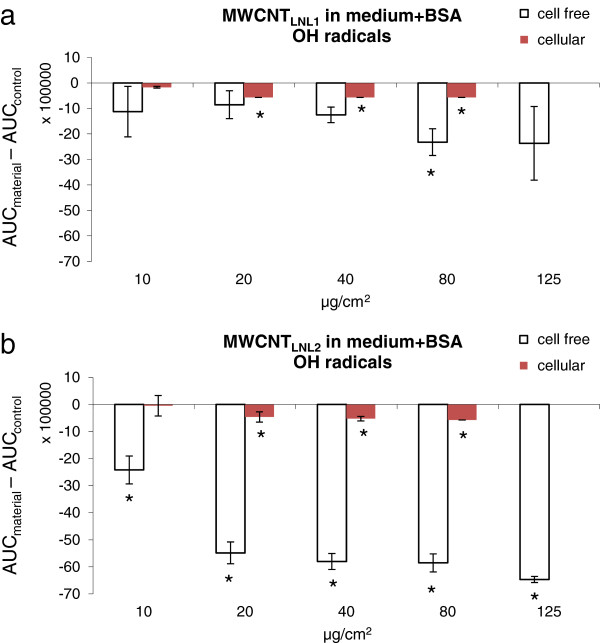
**Intensity of **^**•**^**OH formation by a) MWCNT**_**LNL1 **_**and b) MWCNT**_**LNL2 **_**in medium + 0.6 mg/ml BSA in cell free and cellular settings.** Columns represent the peak surface, i.e. area under curve (AUC), obtained by double integration of the DMPO peaks induced by the materials (AUC_material_) after subtracting that of the control (AUC_control_; ±SD).

In the cell-free settings, the formation of the unique radical seemed to depend on BSA or medium-related proteins for MWCNT_LNL1_, but not for MWCNT_LNL2_. On the other hand, MWCNT_LNL2_ had a stronger ^•^OH scavenging ability which was slightly reduced (p < 0.02) by the addition of BSA in buffer, but not in the cell culture medium (Figure 3). Furthermore, MWCNT_LNL2_ showed a stronger ^•^OH scavenging ability in medium, while MWCNT_LNL1_ only scavenged ^•^OH in buffer (Figure 3). In cellular settings, however, the ^•^OH scavenging ability of both materials was at the same level (Figure 9). Due to the structural and physicochemical complexity of CNTs, we must conclude that extremely well-characterized materials are required to get a clear understanding of the roles of the different properties and characteristics. The presence of iron impurities (as well as other transition metals) has for long been known to be able to induce ROS. Material research has also shown that both graphitic nano-onions [43] and amorphous carbon impurities [44], which both are typical impurities in certain production methods, can play a very significant role increasing the so-called heterogeneous electron transfer rate of MWCNT. Increased sidewall defects in CNT should also increase the electro-chemical reactivity as well as their ability for co-valent functionalization. However, there are several different types of such defects ranging from simple atom vacancies to complex topological re-organizations of the hexagonal lattice [45], which may not all result in similar reactions. As the iron content has been associated with a ROS formation capacity of CNT, structural defects in MWCNT have been associated with ROS scavenging in cell-free systems as well as with lung toxicity responses such as inflammation and genotoxic effects [3]. The observation of materials with scavenging abilities causing toxicological effects is in contrast with the general assumption that the ability of a material to generate, not scavenge, free radicals correlate with its inflammogenicity and genotoxicity. However, the authors [3] speculated whether the scavenging effect and the toxicity are just two unrelated features resulting from the structural defects in the MWCNTs. Indeed, CNTs have previously been shown to scavenge the formation of ROS in cell-free settings, but to induce biologically generated ROS in cellular settings [46]. Furthermore, a recent study reported that structural defects such as dangling bonds are not the only reason for scavenging and electron transfer on the CNT surface [47]. The current study suggests that the effect may, at least in part be associated with the formation of new long-lived radicals. Here, we were not able to see an induction of ^•^OH in cellular settings after 30 min of exposure; although, the scavenging effect was lower in the cellular than in the cell free settings (Figure 9). However, most of the studies reporting ROS formation in cells after exposure to CNTs have applied longer exposure times. For example, ROS and malondialdehyde formation as well as decreased catalase and glutathione activity were reported in human lung cancer cells (A549) following 6-72 h exposures to 0.5-100 μg/ml MWCNTs (Ø 5-20 nm, length 0.3-2 μm) and significant induction of ROS formation was only seen at two doses (10 and 50 μg/ml) [48]. Another group reported increased lipid peroxidation and decreased intracellular glutathione in human embryonic kidney cells following a 48-h exposure to 3-300 μg/ml MWCNTs (Ø 60-80 nm) [49]. In BEAS 2B cells, the level of malondialdehyde DNA adducts (M_1_dG adducts) was shown to increase after 48-h (5 μg/cm^2^) and 72-h (10 and 40 μg/cm^2^) treatments with short SWCNTs (Ø 2 nm, length 1-5 μm), while M_1_dG adducts were decreased after a 72-h treatment with short MWCNTs (40–160 μg/cm^2^, Ø 10-30 nm, length 1-2 μm) [50]. In human mesothelial MeT-5A cells, the short SWCNTs were shown to elevate the level of M_1_dG DNA adducts (1, 5, 10 and 40 μg/cm^2^) after 48 h, but both the short SWCNTs (5-20 μg/cm^2^) and the short MWCNTs (5–160 μg/cm^2^) decreased M_1_dG adduct level after 72 h [50].

BSA has been reported to be one of the best biological surfactants for dispersion of CNTs. As shown by Elgrabli et al. [26] BSA alone did not modify biological responses such as cell viability *in vitro* and inflammatory response *in vivo* compared with saline solution, and CNTs dispersed in BSA altered cellular viability *in vitro* in a similar manner as CNTs dispersed in saline, but showed a better reproducibility, which was probably explained by better dispersion homogeneity in the presence of BSA than without it. Furthermore, BSA did not alter the individual structure of the CNTs, as judged by TEM. Instead BSA was adsorbed to CNT by van der Waals forces. It can be assumed that this type of physisorption also occurs in the lungs after inhalation of the nanomaterials, since serum proteins such as albumin are abundant in pleural fluid and pulmonary surfactant [26,51,52]. Albumin has been shown to act as an antioxidant and structural alteration of the protein, causing changes in its redox potential, has been related to pathological conditions such as inflammation in humans [37]. CNTs have been shown to, not only bind albumin, but also induce secondary and tertiary structural changes in the protein, which indicates that BSA dispersion of CNTs used *in vitro* may have biological relevance [53,54]. On the other hand, pulmonary surfactant proteins A and D have been shown to bind selectively to double-walled CNTs, indicating that the effects of pulmonary surfactant on the radical-generating/scavenging ability of MWCNTs should also be studied [55].

## Conclusions

The results obtained in this study indicate that the specific ROS formation and associated material sedimentation rates, which are linked to primary particle and agglomerate/aggregate size, affect the cytotoxicity of fibres, MWCNTs and Printex 90 carbon black in BEAS 2B cells; MWCNTs with larger, open-structured agglomerates/aggregates and faster sedimentation rates show stronger cytotoxicity. Furthermore, both cell culture medium and BSA have an influence on scavenging and production of ^•^OH radicals by MWCNTs, carbon black, asbestos and glass wool. Finally a unique, yet unidentified, radical formed by long, needle-like MWCNTs was identified. The radical is dose-dependently induced in both cell-free and cellular settings (Figure 8). It can be speculated that this radical is involved in the adverse effects of this type of MWCNTs, since it coincides with the strong cytotoxicity of the two MWCNTs producing this radical and three less cytotoxic (and less pathogenic as described in the Introduction) long, tangled or short MWCNTs do not show the formation of such a radical.

It is important to keep in mind, however, that the results obtained in this study should not, as such, be translated to the *in vivo* situation. The relevance of large agglomerates during inhalation can be questioned, and the limitations of *in vitro* studies need to be considered. Furthermore, physicochemical characteristics needs to be understood and controlled in an even greater detail to understand the specific role of carbon and transition metal impurities as well as the structural defects in the CNT on the formation and scavenging of radicals in biological systems. Also, potential chemical reactions between CNTs and DMPO need to addressed, as previously indicated by Tsuruoka et al [47]. Another subject for future studies are the time-dependent effects, since the protein-binding to nanomaterials may be temporary and degradation of the protein corona (e.g. in the presence of cells) may cause changes in radical formation/scavenging [56]. Nevertheless, our study shows that (i) the different radical formation/scavenging properties of MWCNTs should be taken into consideration when studying oxidative stress by MWCNTs *in vitro*, (ii) BSA influences radical formation/scavenging and (iii) long needle-like MWCNTs may produce unidentified free radicals, which may very well have physiological relevance during human exposure.

## Methods

### Test materials and their characterizations

Commercially available MWCNT_LNL1_ (MWCNT-XNRI-7 from Mitsui & co., Ltd., Tokyo, Japan [Lot# 05072001 K28]; sub-sampled at NRCWE with the code NRCWE-006), MWCNT_LNL2_ (NM-401 from the OECD Working Party on Manufactured Nanomaterials distributed via the European Joint Research Centre [JRC]), MWCNT_LT_ (MWCNT 8-15 nm OD from Cheap Tubes, Inc., Brattleboro, USA; sub-sampled at NRCWE with the code NRCWE-007), MWCNT_SP_ (Baytubes C 150 HP from Bayer Material Science, Leverkusen, Germany), MWCNT_SNP_ (NM-400 from the OECD Working Party on Manufactured Nanomaterials distributed via the JRC) and nano-sized carbon black (Printex 90 from Evonik Industries AG, Essen, Germany) were used. Standard reference crocidolite asbestos was obtained from UICC (Union for International Cancer Control, Geneva Switzerland) and MMVF-10 glass wool was kindly provided by Dr David Brown (School of Life Sciences of the Heriot-Watt University, Edinburgh, United Kingdom).

**Figure 10:**
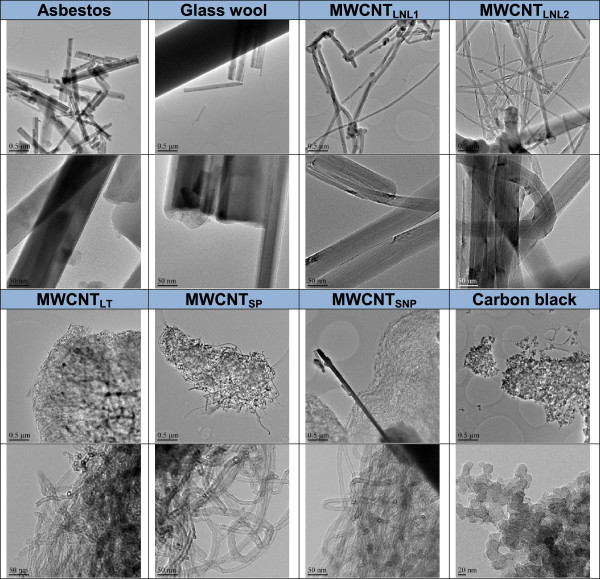
**TEM figures of the test materials.** Shown in two magnifications (the measure bar is 0.5 μm in the upper images and 50 nm in the lower images).

The size and morphology of the materials, as shown in Figure 10, were characterized with TEM (Jeol JEM 2010 TEM, Tokyo, Japan). Samples were prepared by dispersing them into ethanol, and a drop of dispersion was placed onto an amorphous carbon foil copper grid. Elemental analysis of the materials was carried out by energy dispersive spectroscope ThermoNoran Vantage EDS (Breda, The Netherlands) attached to the Jeol JEM 2010 TEM. The description and characteristics of the test materials are shown in Table 1.

### Preparation of exposure dispersions

Material dispersions for cytotoxicity experiments were prepared by weighing the materials into glass tubes and diluting them to a stock dispersion of 2 mg/ml in cell growth medium (BEGM, Clonetics, Walkerwille, MD, USA) with 0.6 mg/ml BSA (Sigma-Aldrich, Steinheim, Germany) and sonicating for 20 min at 37°C using a bath sonicator (Branson 2200, 40 kHz). The stock dispersion was further serially diluted to obtain the final dispersions of 5-350 μg/cm^2^ (corresponding to 19-1330 μg/ml).

For ESR experiments, material dispersions were prepared by weighing the materials into glass tubes and diluting them to a stock dispersion of 1-2 mg/ml in Hank´s balanced salt solution (HBSS; GIBCO BRL) or BEGM with or without 0.6 mg/ml BSA, followed by sonication for 20 min at 37°C using a bath sonicator (Branson 2200, 40 kHz). The stock dispersion was further diluted to 1 mg/ml (for cell-free experiments) or serially diluted to 2-640 μg/ml final dispersions (for cell-free and cellular experiments; representing 0.25-80 μg/cm^2^ in the cell cultures) in HBSS or BEGM with or without 0.6 mg/ml BSA and sonicated a second time for 20 min at 37°C just before ESR measurements or cell exposures. For cell experiments, old medium was carefully removed and replaced with new medium containing the final dispersions of the materials tested.

Dispersions were prepared freshly on the same day and sonicated within 30 min before their application to cells for cellular ESR measurements and before cell-free ESR measurements.

### Characterization of the nanomaterials in exposure medium

A Malvern Nano ZS (Malvern Inc., UK) dynamic light scattering (DLS) instrument equipped with a 633-nm He-Ne laser was used to characterize the hydrodynamic size distributions of two representative dispersions (1330 and 38 μg/ml) in the *in vitro* exposure media (BEGM + 0.6 mg/ml BSA). For sizing, app. 0.7 ml was added into disposable 1 ml standard polystyrene cuvette. Thermal equilibrium time was set to 2 minutes, and analysis was started ca. 3-5 minutes after dispersion following the protocol used for *in vitro* cytotoxicity testing. A refraction index of 2.02 and an optical absorption of 2.00 was used for the materials for the calculations along with standard optical indices for water and a dynamic viscosity of 0.95 cP (0.95 ± 0.04 cP; n = 3). The viscosity of the BEGM + 0.6 mg/ml BSA was measured using an AND Vibro Viscometer Model SV-10 (A&D Company, Ltd, Tokyo, Japan) and 10 ml flow-cell cuvettes at 37°C, corresponding to the analytical conditions for DLS analysis. The temperature was ensured using recirculated water conditioned in Polyscience AD07R-20 Refrigerating/Heating Bath (Polyscience, IL, USA). Initial size-distribution measurements were completed based on ten (stock dispersions) or six (exposure concentrations) repeated analyses using an automated optimization procedure given by the Malvern Software. Slides for optical microscopy and grids for TEM were prepared from the batch dispersions (1.333 and 0.038 mg/ml) to support the interpretation of the DLS data.

The sedimentation was assessed by measurements of the dispersion in the 0.7-ml cuvettes for up to 48 h at the exposure concentrations 1330 and 38 μg/ml using the scattered intensity as a relative scale for the amount of test material in suspension. The measurements were generally completed at a fixed measurement interval of 15 min after the initial six size-distribution measurements. The automatic settings for the initial measurements were fixed for the subsequent sedimentation analysis.

Optical microscopy was applied for qualitative assessment of the dispersion with focus on the presence of large agglomerates and aggregates. Optical micrographs were obtained using a Nikon DS-Fi2 (Tokyo, Japan) digital camera attached to a Leica DMIL (Wetzlar, Germany) optical transmission light microscope. Image acquisition was made using the Nikon DS-U3 Digital Camera Control Unit software (vs. 1.10). Field of view at maximum magnification was 281 × 210 μm. Samples were made by placing a droplet of the suspensions onto a glass-slide and covered with a cover-glass. Analyses were made immediately after starting the DLS analysis to avoid drying of the medium.

The nature of the dispersed test materials in exposure medium was assessed using TEM. Samples were prepared by placing a drop of dispersion onto an amorphous carbon foil 200 mesh copper grid. Only samples from the 1330 μg/ml dispersion were fully analysed in TEM and are shown in Figure 2. Estimation of the number of agglomerates in the dispersion was done by calculating the number of separate agglomerates from the image area of 2000 μm^2^.

### Cell culture

Transformed human bronchial epithelial BEAS 2B cells, exhibiting an epithelial phenotype [57], were obtained from the American Type Culture Collection through LGC Promochem AB (Borås, Sweden). The BEAS 2B cells were grown in serum-free BEGM medium at 37°C in a humidified atmosphere of 5% CO_2_.

### Cytotoxicity

Twenty-thousand cells were plated on 24-well plates (culture area 1.9 cm^2^/well; culture medium volume 0.5 ml/well) and grown to semiconfluency (2-3 days). The cells were exposed to 500 μl per well of ultrasonicated dispersions of MWCNT_LNL1_, MWCNT_LNL2_ MWCNT_LT_, MWCNT_SP_, MWCNT_SNP_, carbon black, asbestos and glass wool for 4, 24 and 48 h at doses 5, 10, 50, 80, 100, 200, 250, 300 and 350 μg/cm^2^ (corresponding to 19, 38, 190, 304, 380, 760, 950, 1140 and 1330 μg/ml). For asbestos, also 1, 2, 4, 8, 12 and 16 μg/cm^2^ (corresponding to 3.8, 7.6, 15.2, 30.4, 45.6 and 60.8 μg/cm^2^) were tested. Untreated controls were included at all time points. All the doses were tested with 4 replicates (2 separate experiments, each with 2 parallel samples).

Cytotoxicity was measured using the trypan blue dye exclusion technique (after collecting cells by trypsination), i.e. by manually counting the number of living (unstained) cells using phase-contrast microscopy. Cell number was expressed as the percentage of viable cells in the treated cultures in comparison with the control cultures. These assays reflect all treatment-related effects (necrosis, cell cycle delay, and apoptosis) that reduce the number of viable cells. Half maximal inhibitory concentration (IC_50_) was calculated by fitting the data to a logarithmic trend line with the formula: *y* = *a* ∗ ln(*x*) + *b* (where a = slope and b = y-intercept).

### ESR spectroscopy

For cell-free ESR experiments, 200 μl of nanomaterial dispersions in buffer or BEGM with or without BSA (1 mg/ml or in the case of MWCNT_LNL1_ and MWCNT_LNL2_ 80, 160, 320 and 640 μg/ml corresponding to 10, 20, 40 and 80 μg/cm^2^ in the cellular settings) was incubated with 1 mM H_2_O_2_ and 50 mM 5,5-dimethyl-1-pyrroline N-oxide (DMPO; Sigma-Aldrich, Munich, Germany) in a CO_2_ incubator at 37°C for 30 min, as described previously [58]. DMPO reacts with oxygen-, nitrogen-, carbon- and sulfur-centered radicals and “traps” them to prevent their degradation before measurement with ESR. The trapping by DMPO results in unique ESR spectra for each type of free radical. Positive controls for ^•^OH formation and scavenging were performed using iron(II) sulphate (FeSO_4_) and the iron chelator deferoxamine (DFO) Additional file 1: Figure S10). For each sample, a 100-μl glass capillary (Brand, Wertheim, Germany) was filled with the suspension and sealed with wax.

For cellular ESR experiments 150 000 or 100 000 BEAS-2B cells were plated out and grown to semiconfluency (for 2-3 days) in 8-cm^2^ culture dishes (BD Falcon, New Jersey, USA). The cells were exposed to 1 ml of MWCNT_LNL1_ and MWCNT_LNL2_ dispersions to reach final doses of 10, 20, 40 or 80 μg/ cm^2^ (corresponding to 80, 160, 320 and 640 μg/ml) or 2 and 10 μg/ cm^2^ for asbestos and glass wool, in BEGM with BSA for 30 min (in the case of asbestos-exposures also for 1, 2 and 4 h). The spin trapping agent DMPO (50 mM) was added for the last 30 min, i.e. at the same time as the exposure solution for the 30 min exposures. The cells were subsequently harvested by scraping and homogenized by pipetting. For each sample, a 100-μl glass capillary (Brand, Wertheim, Germany) was filled with the suspension and sealed with wax.

ESR measurements were carried out as previously described [58]. Briefly, after sealing, the capillary was immediately placed in the resonator of the ESR spectrometer. During the exposure and measurement of the samples, light exposure was kept to a minimum. ESR spectra were recorded at room temperature on a Bruker EMX 1273 spectrometer equipped with an ER 4119HS high-sensitivity resonator and 12-kW power supply operating at X band frequencies. The spectra were quantified by peak surface measurements (area under curve; AUC) through double integration of the ESR spectrum using the WIN-EPR spectrum manipulation program (Bruker BioSpin, Wormer, the Netherlands). Spectra were created at identical intensity scales to enable visual comparison of the different conditions in each figure. All experiments were performed with three replicates, and statistical analysis was performed using an unpaired, two-tailed *t*-test with a 95% confidence interval to examine whether the induction of radical formation was significantly different in the samples with test materials or in the treated cells as compared with the controls. For visualization in column graphs, the mean of the replicate controls (AUC_control_) was subtracted from the mean of the replicate samples (AUC_material_). Linear regression analysis was used to examine linear dose-response of radical formation.

## Competing interests

The authors declare that they have no competing interests.

## Authors’ contributions

PN designed and carried out the ESR studies, coordinated the cytotoxicity studies, interpreted the data, and drafted the manuscript. KAJ and YK designed and conducted the physicochemical characterizations of the dispersions and their sedimentation, while MV performed the microscopy characterizations of the dry and dispersed materials. KAJ also contributed to the interpretation of the data. SS carried out the cytotoxicity studies. JK and JvD consulted during the study and participated in the drafting of the manuscript. JC obtained the test materials, consulted during the study and helped in the drafting of the manuscript. HN coordinated the acquisition of the nanomaterials and the cytotoxicity tests, and substantially contributed to the drafting of the manuscript. JB conceived the study and participated in its design, and helped in drafting the manuscript. All authors read and approved the final manuscript.

## Supplementary Material

Additional file 1 **The dispersibility and sedimentation of nanomaterials in the BEGM + 0.6 mg/ml BSA stock suspensions and exposure medium was assessed by optical microscopy and photon correlation spectroscopy using a Malvern Dynamic Light Scattering Nano ZS equipment.** The size-distributions and behaviour in the exposure media were assessed based on up to 48 h long in situ DLS analysis of average particle size (zeta-size) and temporal evolution of the ratios between the measured and initial intensity of the scattered laser light (I/Io). In this supplemental material, an image of the batch dispersion as well as the temporal evolution in particle size and the I/Io ratio is given for each nanomaterial. These two images are accompanied by a summery text of the optical microscopy and DLS analysis. In each image pair A) shows an optical micrograph of each material dispersed in BEGM + 0.6 mg/ml BSA stock suspension and B) shows the sedimentation and average zeta-size curves for the dispersed material at 0.038 and 1.333 mg/ml (corresponding to 10 and 350 μg/cm2). Click here for file
