# Supplementary material for: Free radical scavenging and formation by multi-walled carbon nanotubes in cell free conditions and in human bronchial epithelial cells
Source: Part Fibre Toxicol. 2014 Jan 18;11:4. doi: 10.1186/1743-8977-11-4 (PMC3933237; doi:10.1186/1743-8977-11-4)
Supplement: Additional file 1 — The dispersibility and sedimentation of nanomaterials in the BEGM + 0.6 mg/ml BSA stock suspensions and exposure medium was assessed by optical microscopy and photon correlation spectroscopy using a Malvern Dynamic Light Scattering Nano ZS equipment. The size-distributions and behaviour in the exposure media were assessed based on up to 48 h long in situ DLS analysis of average particle size (zeta-size) and temporal evolution of the ratios between the measured and initial intensity of the scattered laser light (I/Io). In this supplemental material, an image of the batch dispersion as well as the temporal evolution in particle size and the I/Io ratio is given for each nanomaterial. These two images are accompanied by a summery text of the optical microscopy and DLS analysis. In each image pair A) shows an optical micrograph of each material dispersed in BEGM + 0.6 mg/ml BSA stock suspension and B) shows the sedimentation and average zeta-size curves for the dispersed material at 0.038 and 1.333 mg/ml (corresponding to 10 and 350 μg/cm2). [file 1743-8977-11-4-S1.doc]

# Additional file 1

# Free radical scavenging and formation by multi-walled carbon nanotubes in cell free conditions and in human bronchial epithelial cells

Penny Nymark^a*^, Keld Alstrup Jensen^b^, Satu Suhonen^c^, Yahia Kembouche^b^, Minnamari Vippola^c, d^, Jos Kleinjans^a^, Julia Catalán^c^, Hannu Norppa^c^, Joost van Delft^a^ , Jacob Jan Briedé^a^

^a^ Department of Toxicogenomics, Maastricht University, Maastricht, The Netherlands

^d^ Danish Centre for Nanosafety, National Research Centre for the Working Environment, Copenhagen, Denmark

^c^ Nanosafety Research Centre and Systems Toxicology, Finnish Institute of Occupational Health, Helsinki, Finland

^d^ Department of Materials Science, Tampere University of Technology, Tampere, Finland

* Corresponding author

The dispersibility and sedimentation of nanomaterials in the BEGM + 0.6 mg/ml BSA stock suspensions and exposure medium was assessed by optical microscopy and photon correlation spectroscopy using a Malvern Dynamic Light Scattering Nano ZS equipment. The size-distributions and behaviour in the exposure media were assessed based on up to 48 h long in situ DLS analysis of average particle size (zeta-size) and temporal evolution of the ratios between the measured and initial intensity of the scattered laser light (I/Io).

In this supplemental material, an image of the batch dispersion as well as the temporal evolution in particle size and the I/Io ratio is given for each nanomaterial. These two images are accompanied by a summery text of the optical microscopy and DLS analysis. In each image pair A) shows an optical micrograph of each material dispersed in BEGM + 0.6 mg/ml BSA stock suspension and B) shows the sedimentation and average zeta-size curves for the dispersed material at 0.038 and 1.333 mg/ml (corresponding to 10 and 350 µg/cm^2^).


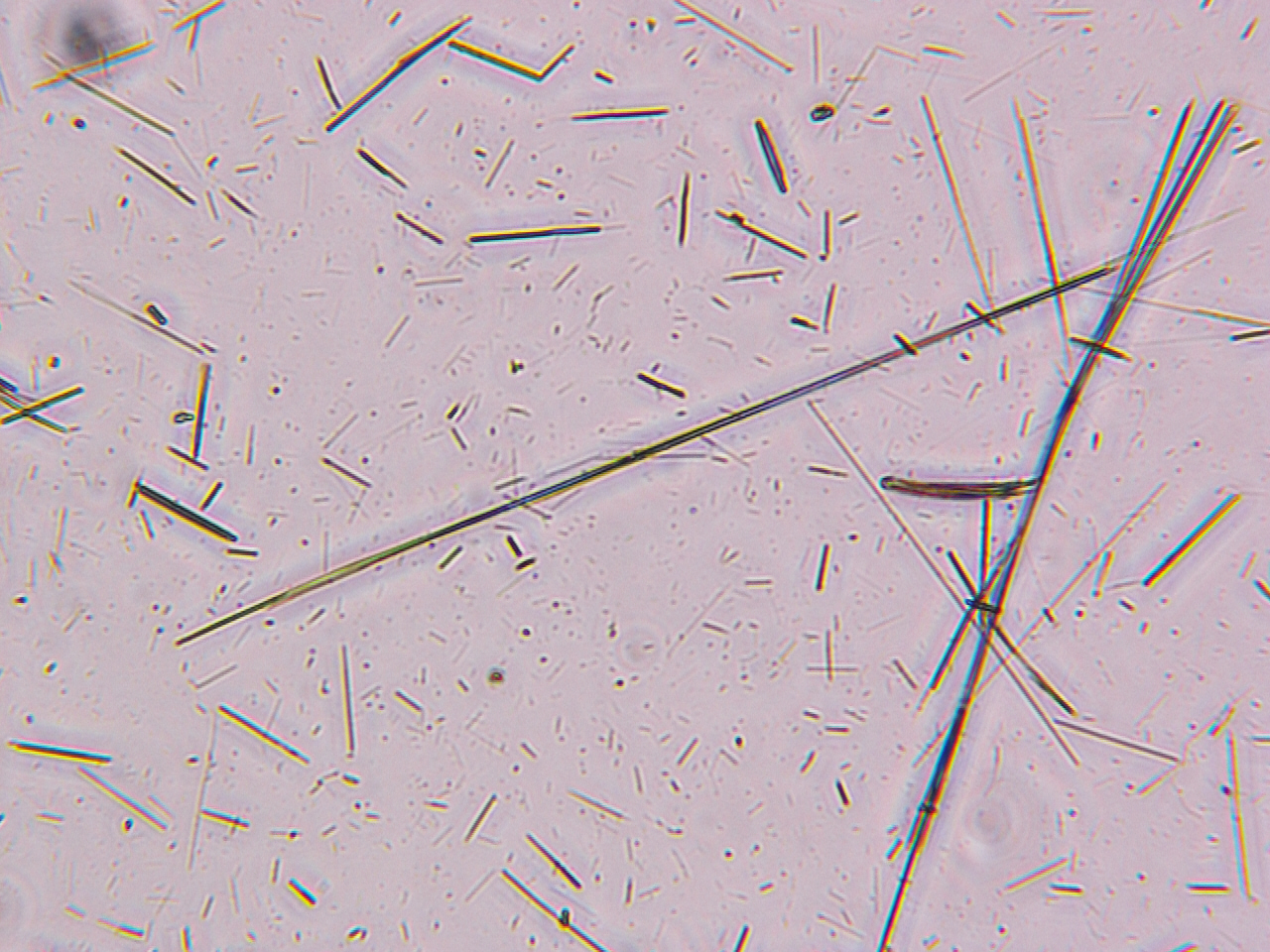


100 µm

**A)**

**B)**

**Figure S1:** **Asbestos A)** . The fibres have very large variations in aspect ratio. The longest observed fibres were about 200 to 800 µm long, but these were rare. Most fibres were shorter than 5-10 µm. **B)** The I/Io curve indicated rapid initial sedimentation at both concentrations followed by a long period with slow continuous sedimentation. The average zeta-size of the dispersed particles varies to some degree with concentration. The fast initial drop in size indicates fast sedimentation of the largest fibres. However, photon correlation spectra indicate the presence of large (out of range) particles during most of the measurement duration making the size-distributions highly polydispersed and mostly not suitable for sizing.


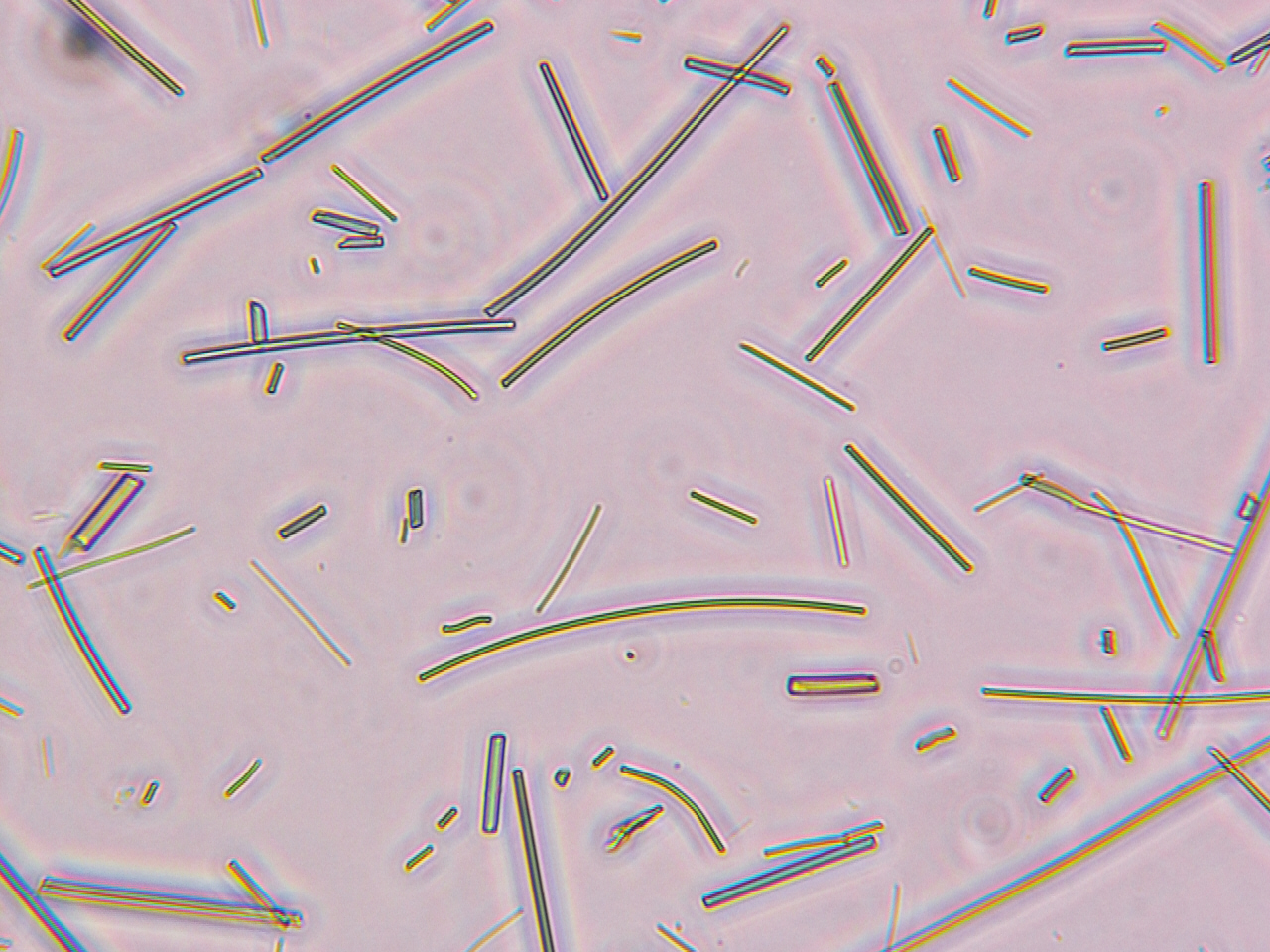


100 µm

**A)**

**B)**

**Figure S2:** **Glass wool A)** The fibres have very large variations in aspect ratio. The longest observed fibres were approximately 100 to 200 µm long, but the total range was very wide possibly reaching into the sub-µm range. **B)** The I/Io curve indicates rapid initial sedimentation followed by an extended period with slower sedimentation at both concentrations. Size and intensity data suggest that most of the material is deposited after about 1100 min. The average zeta-size of the dispersed particles is around 1 µm and initially drops quickly due to fast sedimentation of largest fibres. The photon correlation spectra show presence of large (out of range) particles and highly polydispersed size-distributions in all measurements. For the 0.038 mg/ml suspension, the 48-hour incubation ends with accumulation of particles at the vial bottom resulting in the observed increase in the I/Io and the average zeta-size at about 2800 min.


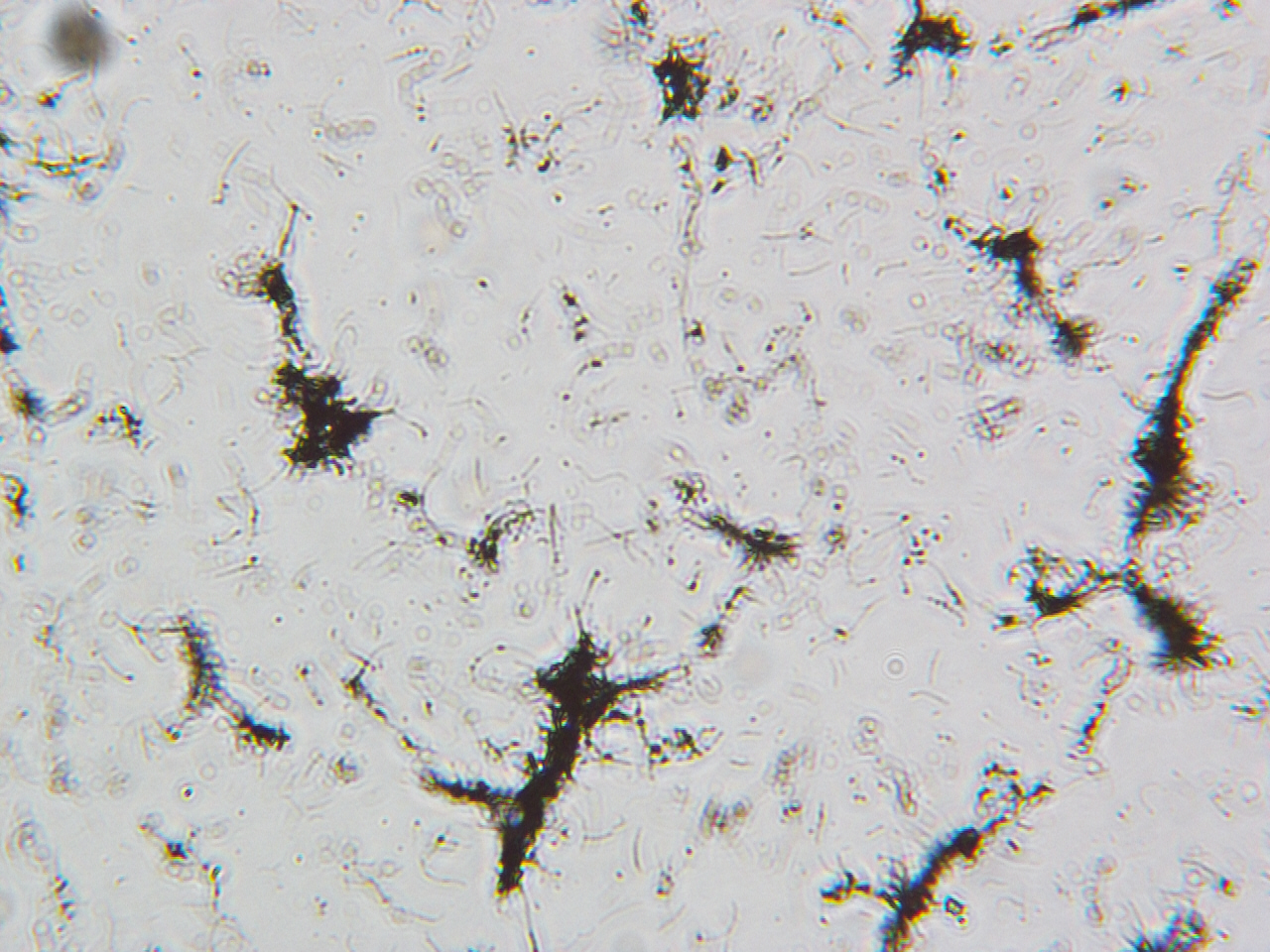


100 µm

**A)**

**B)**

**Figure S3: MWCNT_LNL1_ A)** The suspension consists of a mixture of individual tubes or tube-bundles and small particles as well as larger irregularly shaped CNT aggregates/agglomerates dispersed into the medium. Individual CNT protrude from these aggregates. The maximum lengths of the CNT are on the order of 10 µm. **B)** The evolution of both curves indicates rapid sedimentation at both concentrations reaching approximately 0.1 I/Io at 1000 minutes. The photon correlation spectra show presence of large (out of range) particles and highly polydispersed size-distributions in all measurements.


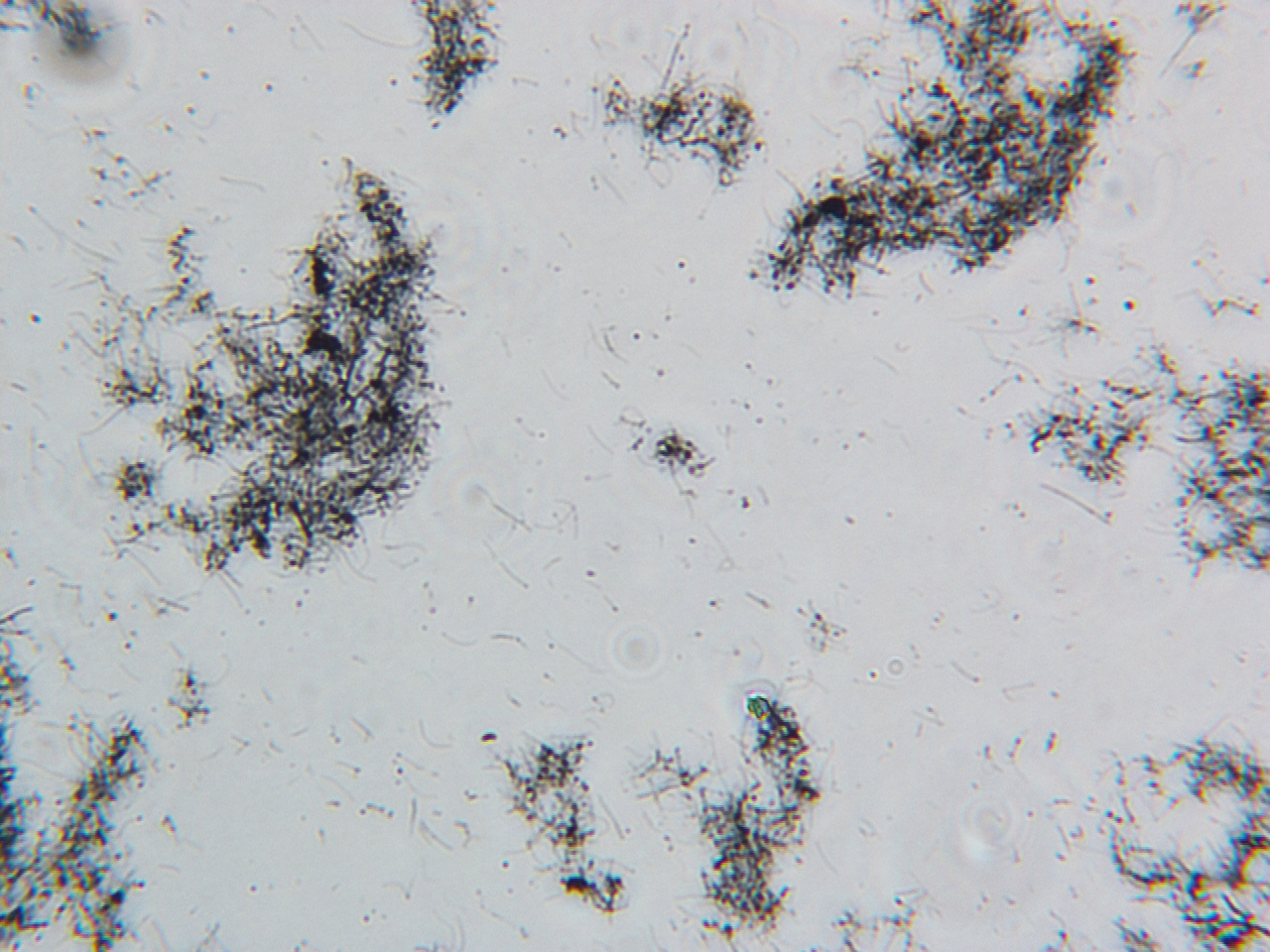


100 µm

**A)**

**B)**

**Figure S4:** **MWCNT_LNL2_ A)** The suspension consists of a mixture of individual tubes or tube-bundles and small particles dispersed into the medium and larger irregularly shaped and partially open-structured CNT aggregates/agglomerates. Individual CNT protrude from these aggregates. The tube lengths are in the order of 10 to 20 µm. **B)** The evolution of both curves indicate rapid sedimentation at both concentrations reaching approximately 0.1 I/Io at 1000 minutes. The photon correlation spectra show presence of large (out of range) particles and the size-distributions are highly polydispersed in all measurements.


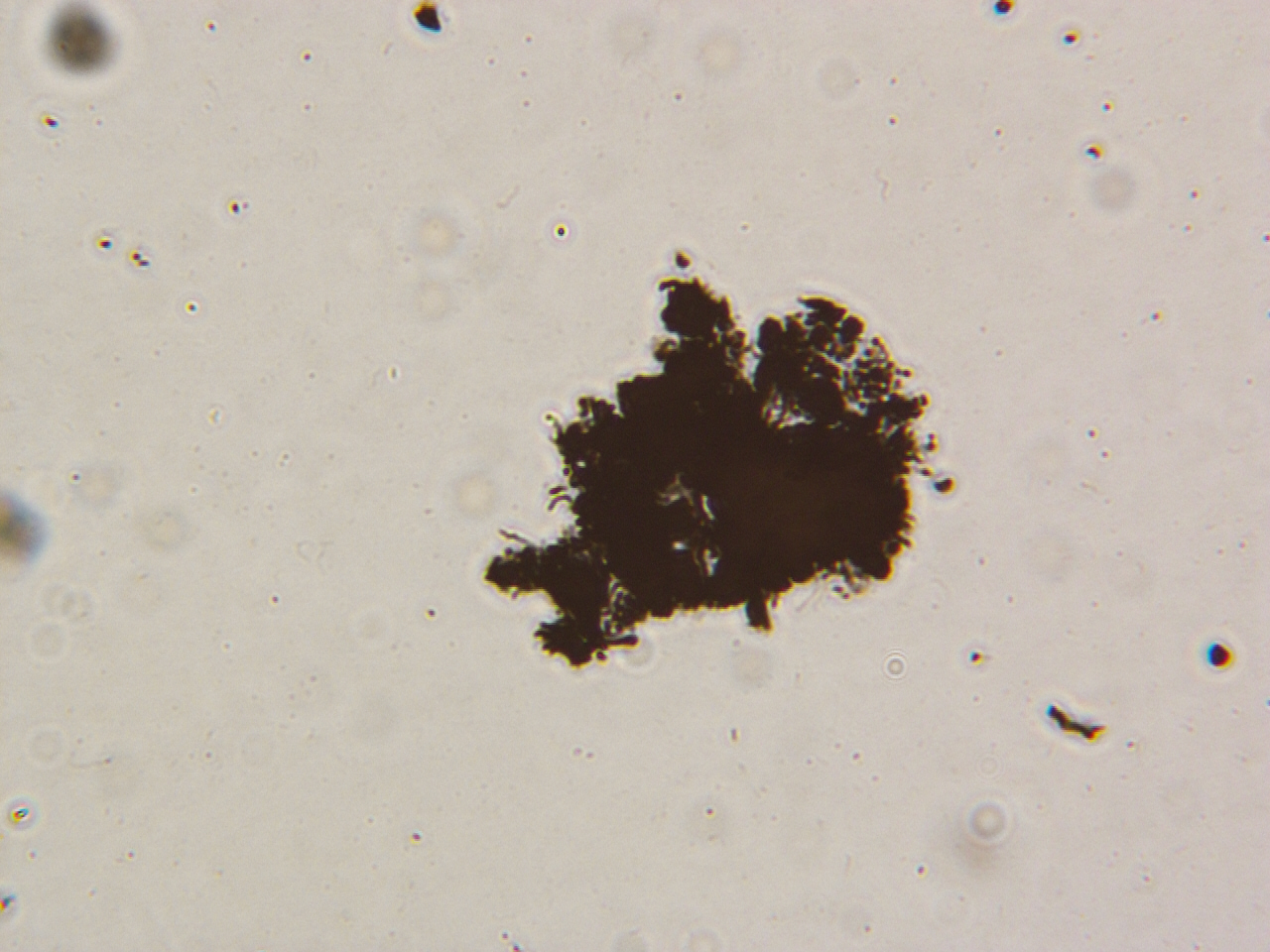


100 µm

**A)**

**B)**

**Figure S5:** **MWCNT_LT_ A)** From the low magnifications of the optical microscope, the dispersion is dominated by dense up to about 100 µm-size aggregates/agglomerates and some single tubes or tube-bundles dispersed into the medium. Some CNTs protrude from the aggregates. **B)** The data indicate rapid sedimentation and episodic particle agglomeration (increase in I/Io) at the vial bottom at both concentrations. Sedimentation is a rather advanced at between 500 and 1000 minutes. The photon correlation spectra show presence of large (out of range) particles and the size-distributions are generally highly polydispersed with volume-size peaks at around 400 nm and 3-5 µm during the whole measurement period. At both concentrations, the average zeta-size increases at the end of the measurement due to aggregate/agglomerate accumulation at the vial bottom.


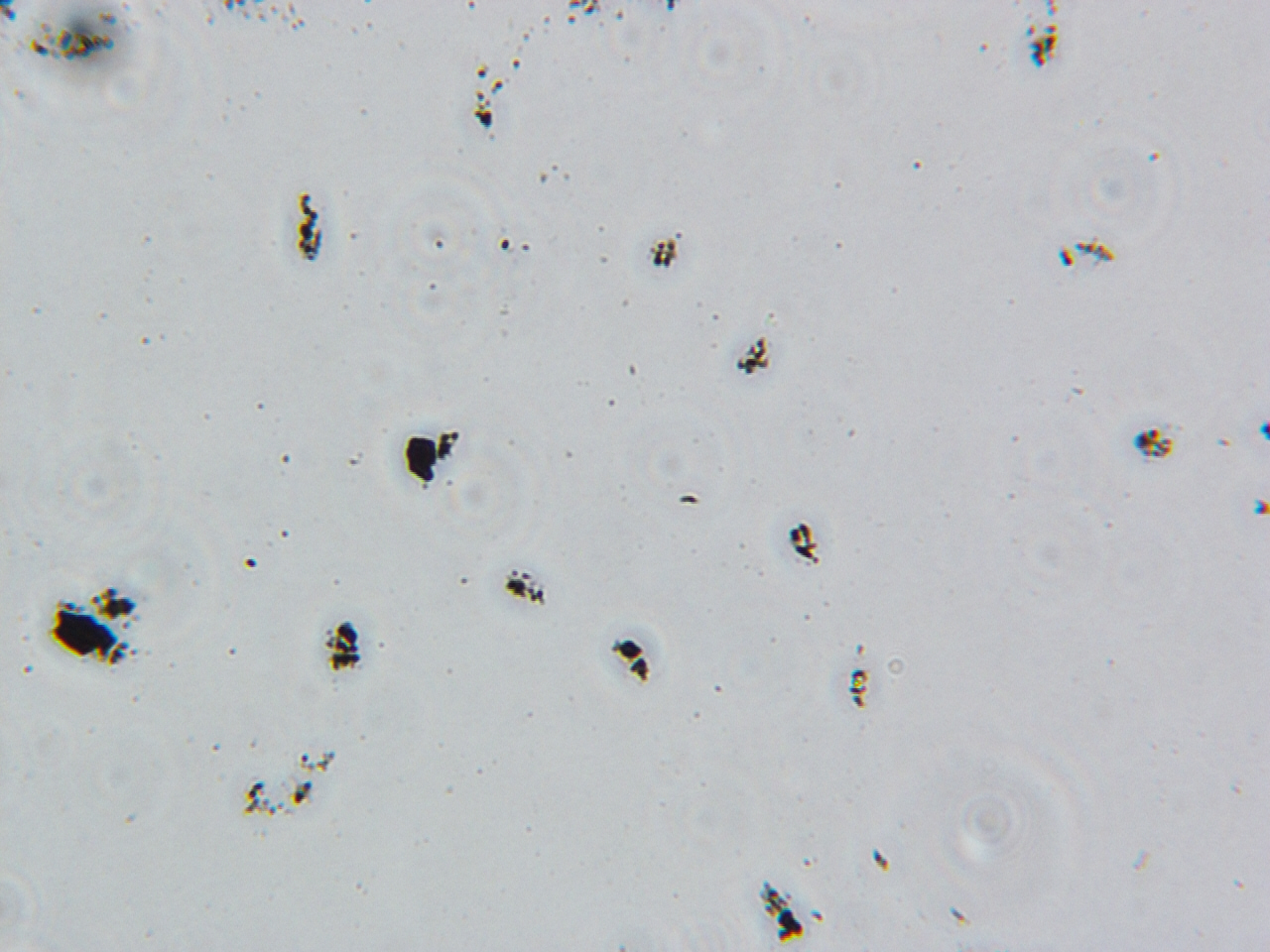


100 µm

**A)**

**B)**

**Figure S6: MWCNT_SP_** **A)** The dispersion is dominated by dense up to about 20 µm-size aggregates/agglomerates. Free CNTs were barely visible and rare. **B)** The initial photon correlation spectra show presence of large (out of range) particles with highly polydispersed size-distributions. After rapid sedimentation of the coarse particles, the size-distribution spectra become measurable and continuously slowly finer with time in both the 1.333 and 0.038 mg/ml dispersions. The valid size-distribution spectra suggest two major modes at 300-400 nm and a coarser in the 3 to 5 µm size-range. The 0.038 mg/ml dispersion becomes unstable and sediments after ca. 2300 minutes with formation of a stratified dispersion.


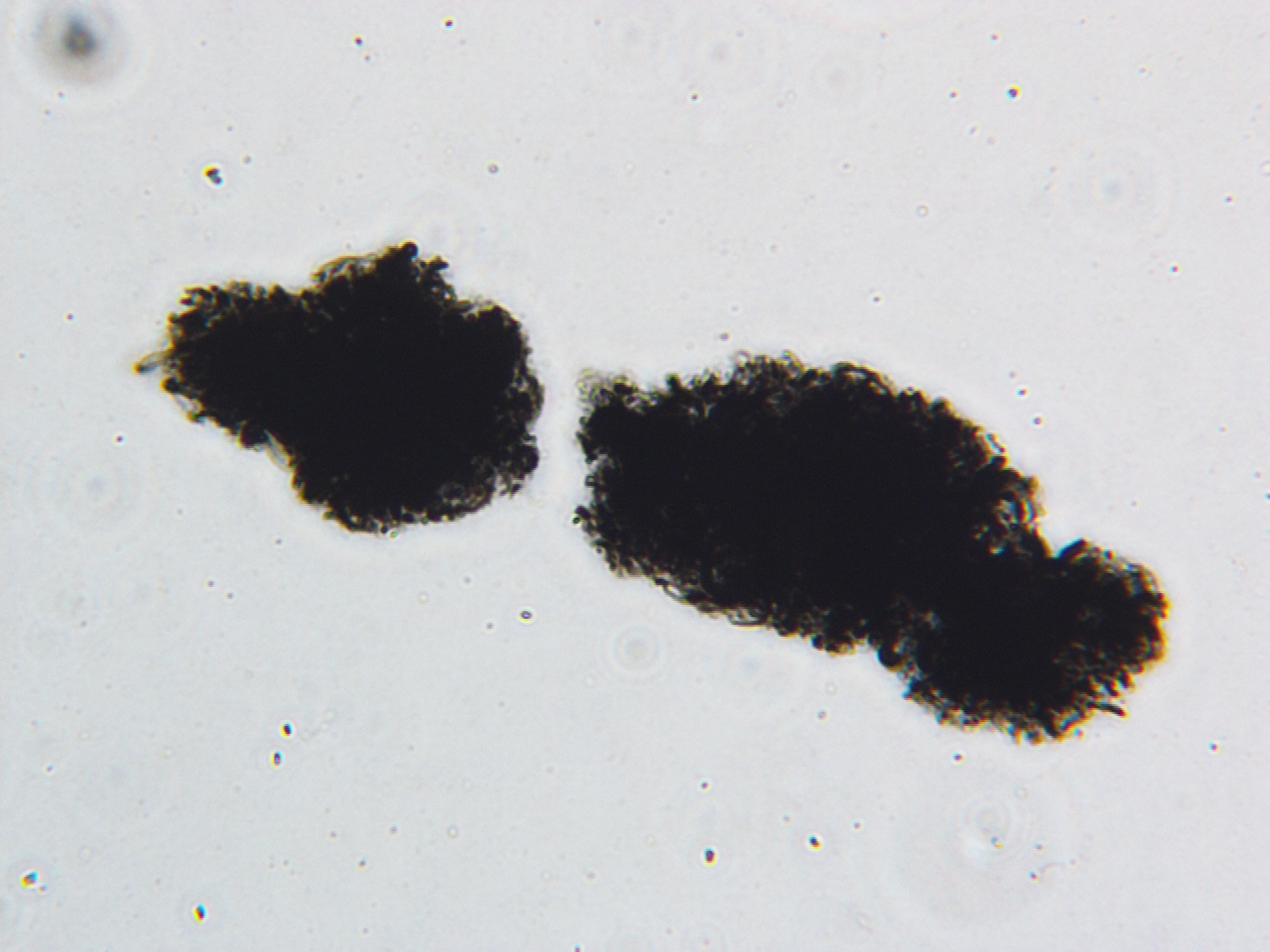


100 µm

**A)**

**B)**

**Figure S7: MWCNT_SNP_** **A)** The dispersion is dominated by dense up to ca. 100 µm-size aggregates/agglomerates. Free CNTs were barely visible and rare. **B)** After rapid settling of the largest aggregates/agglomerates, the photon correlation spectra of the 1.333 mg/ml dispersion give acceptable size-distributions with two major modes at ca. 400 nm and a coarser at ca. 4 µm. Measurements stop at 4 hours into the experiment. The sizing data from 0.038 mg/ml dispersion are questionable always showing presence of large out of range particles in the correlograms and distribution fits and high polydispersivity. However, the I/Io evolution path suggests sedimentation and agglomeration with an episode of particle accumulation at the vial bottom after roughly 250 min measurement.


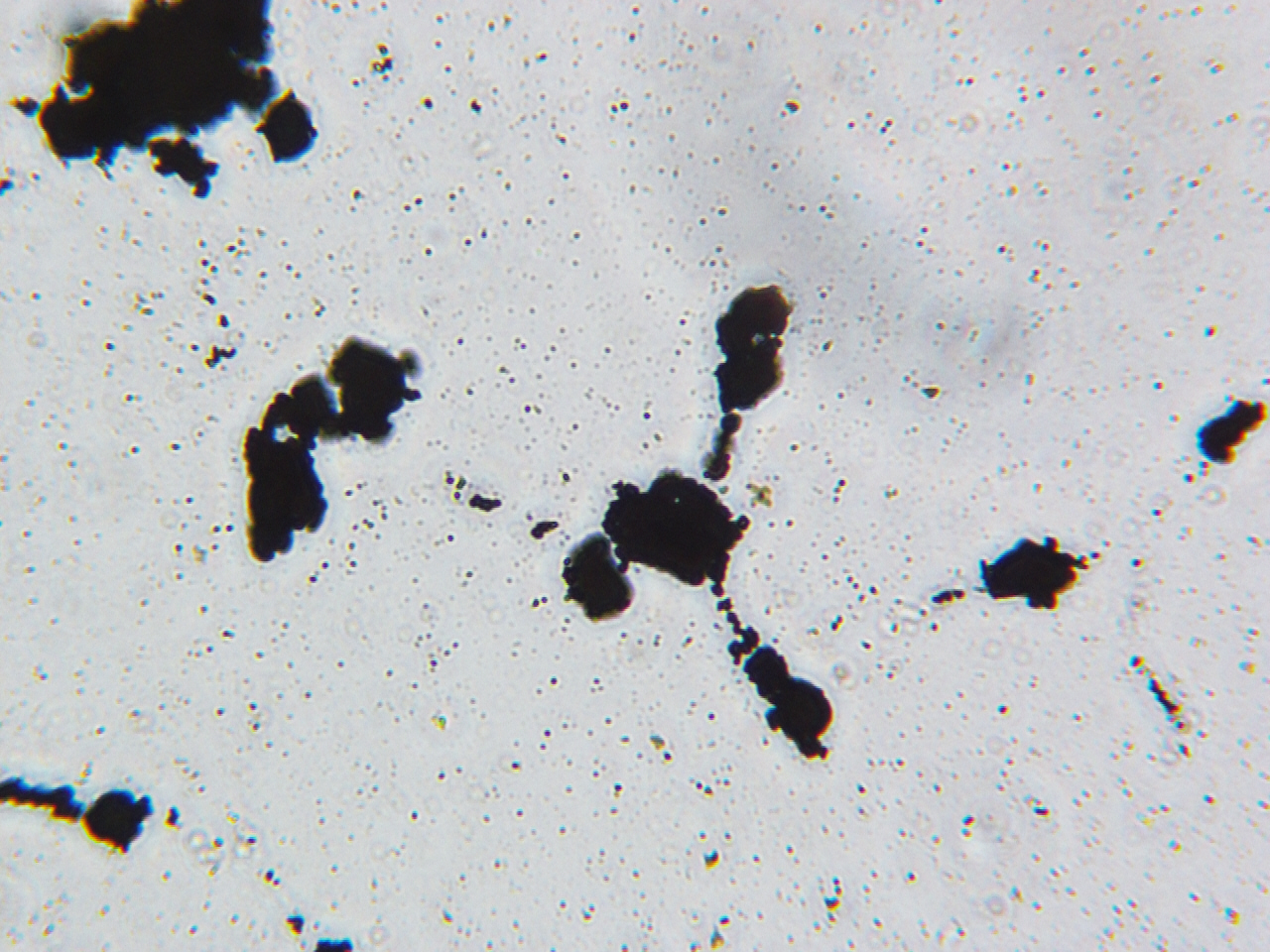


100 µm

**A)**

**B)**

**Figure S8: Carbon black (Printex 90)** **A)** The dispersion consists of dense up to around 50 µm-size aggregates/agglomerates with the majority in sub-µm to µm-size aggregates. **B)** Photon correlation spectra of both the 0.038 and the 1.333 mg/ml dispersion give acceptable size-distributions during the entire measurement period. A few large (borderline or out of range) particles are present in most analyses of the two dispersions. The volume size-distribution spectra suggest that the carbon black dispersions are at least bimodal with initial major size-modes at approximately 400 nm and 5 µm. The sub-µm size-mode decreases to 200-300 nm-size in the 0.038 mg/ml dispersion.


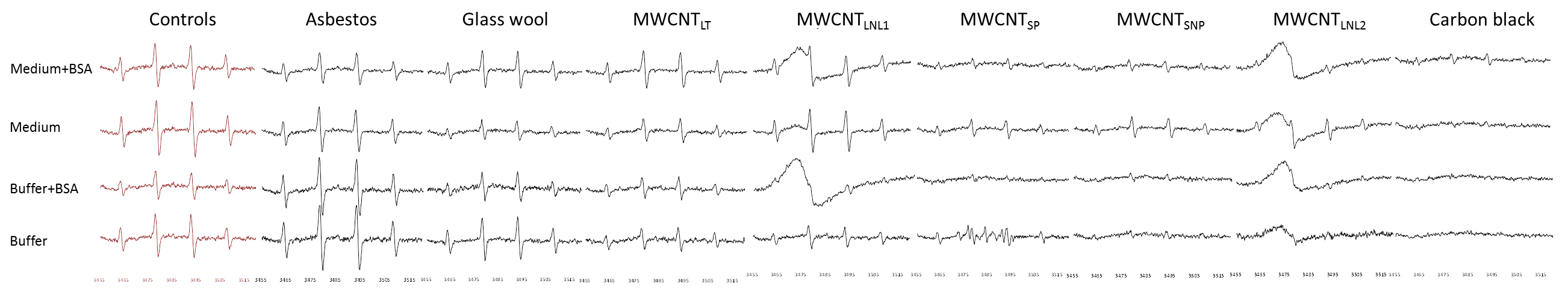


**Figure S9:** ESR spectra of controls (red; buffer or medium with or without BSA) and all 8 test materials (black) in the four different dispersions (buffer and medium with or without BSA). All dispersions contain H_2_O_2_ and DMPO and all spectra are shown at identical intensity scale.


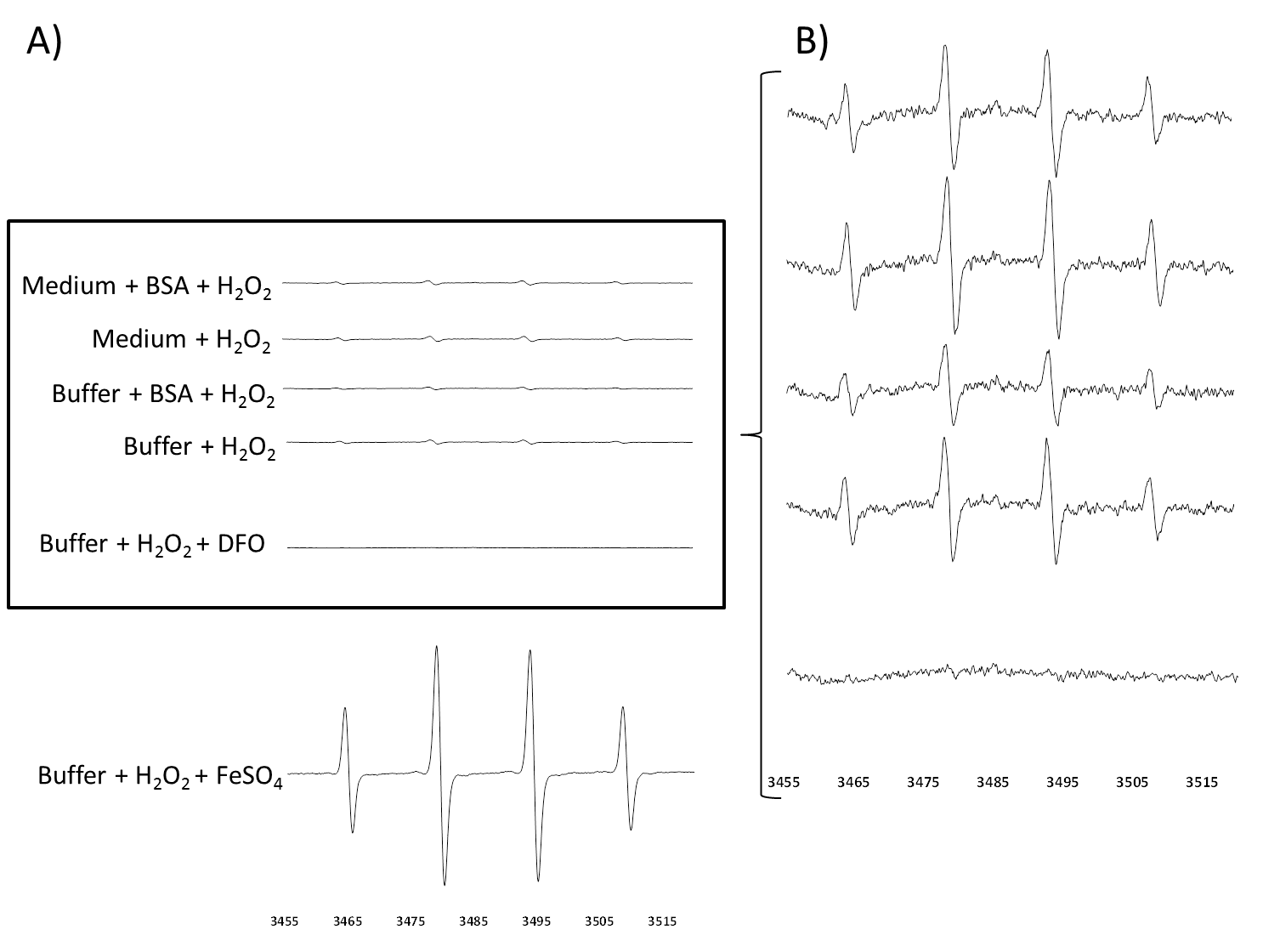


**Figure S10: A)** ESR spectra of the four control suspensions (buffer and medium with or without BSA and H_2_O_2_), a positive control for ^•^OH scavenging (buffer with H_2_O_2_ and the iron chelator deferoxamine [DFO]), and a positive control for ^•^OH generation (buffer with H_2_O_2_ and FeSO_4_). Spectra are shown at identical intensity scale. **B)** Enlarged spectra of the four control suspensions. Spectra are shown at identical intensity scale. All samples contain DMPO.
